# Biokinetics and clearance of inhaled gold ultrasmall-in-nano architectures†

**DOI:** 10.1039/d0na00521e

**Published:** 2020-07-22

**Authors:** Ana Katrina Mapanao, Giulia Giannone, Maria Summa, Maria Laura Ermini, Agata Zamborlin, Melissa Santi, Domenico Cassano, Rosalia Bertorelli, Valerio Voliani

**Affiliations:** Center for Nanotechnology Innovation@NEST, Istituto Italiano di Tecnologia Piazza San Silvestro 12 – 56127 Pisa Italy valerio.voliani@iit.it; NEST-Scuola Normale Superiore Piazza San Silvestro 12 – 56127 Pisa Italy; Translational Pharmacology, Istituto Italiano di Tecnologia Via Morego 30 – 16163 Genoa Italy

## Abstract

Among an organism's entry portals, the respiratory tract is one of the most promising routes for non-invasive administration of therapeutics for local and systemic delivery. On the other hand, it is the subtlest to protect from environmental pollution and microbial occurrences. Here, the biokinetics, distribution, and clearance trends of gold ultrasmall-in-nano architectures administered through a single intranasal application have been quantitatively evaluated. Apart from reaching the lung parenchyma, the (bio)degradable nano-architectures are able to translocate as well to secondary organs and be almost completely excreted within 10 days. These findings further support the clinical relevance of plasmonic nanomaterials for oncology and infectious disease treatment and management. Notably, this investigation also provides crucial information regarding the associated risks as a consequence of the pulmonary delivery of nanoparticles.

## Abstract 2

After inhalation, ultrasmall-in-nano gold architectures accumulate in the lungs, reach secondary organs, and are excreted, encouraging the clinical translation of plasmonic nanomaterials to the management of cancer and infectious diseases.

## Graphical abstract

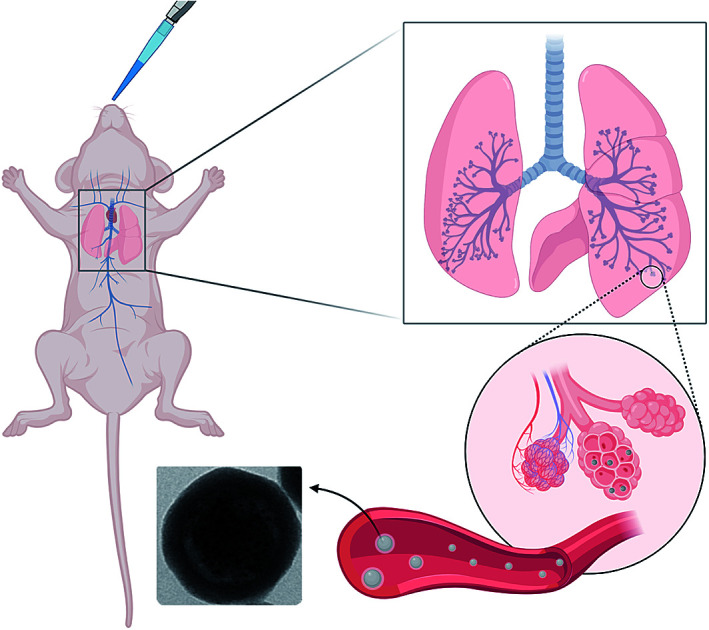

## Introduction

Investigations on the biokinetic behaviours of nanomaterials, including the absorption–distribution–metabolism–elimination (ADME) processes, are essential for their clinical translation and safety evaluation in agreement with the opinions of the Scientific Committee on Emerging and Newly Identified Health Risks (SCENIHR).^1,2^ Nanomaterials can penetrate into organisms by voluntary administration or unintentional contact and through several modes such as injection (intravenous, subcutaneous, and intratumoral), inhalation, ingestion, and dermal contact.^3^ Among these, delivery through inhalation is especially interesting due to the large surface area of the lungs (average 150 m^2^), thin alveolar epithelium, easily permeable membrane, and extensive vasculature, which can allow substantial and rapid absorption of nanomaterials.^4,5^ Moreover, patients can self-administer therapeutics without the need for a trained staff.^4^ The inhalation route may increase local or systemic efficacy and bioavailability of therapeutics as it circumvents metabolic barriers such as the hepatic first-pass effect.^6^ Hence, it is ideal and practical for the delivery of drugs and emerging systems like nanomaterials, in order to treat respiratory ailments like asthma and microbial infections, as well as systemic diseases.^7,8^ Overall, identifying and analysing the biokinetics of nanomaterials in terms of accumulation in the lungs, translocation to secondary organs, and excretion after exposure are crucial from both clinical and environmental perspectives.^9^

In this regard, the nose has an important role in inhalation administration as the primary portal for respiration. It also has olfactory functions and protects the lungs by heating, humidifying and filtering the incoming airstream.^10^ Interestingly, the nasal pathway offers an alternative route for the delivery to the brain, as therapeutics deposited onto the olfactory epithelium can directly translocate to the brain through the olfactory and trigeminal nerve pathways avoiding the strictly selective blood–brain barrier (BBB) and minimizing systemic exposure.^3,10,11^

In this study, the biokinetics, distribution, and clearance trends of gold ultrasmall-in-nano architectures (NAs) administered through a single intranasal application have been quantitatively evaluated. Our findings confirmed the localization of NAs in the lung parenchyma, the translocation of metal nanoparticles to secondary organs, and, following the (bio)degradation of NAs, an almost complete excretion of the metal from the organism within 10 days. Remarkably, NAs are non-persistent noble metal-based nano-architectures of special interest for oncology and infectious disease treatment.^12–15^ Their (bio)degradation to excretable building blocks has already been demonstrated together with their biosafety features at therapeutic concentrations in different vertebrate models.^16–18^ Overall, these findings reinforce the clinical applicability of noble metal-based nanotherapeutics and provide useful evidence on the risks associated with pulmonary exposure to nanomaterials.

## Results and discussion

**Fig. 1:**
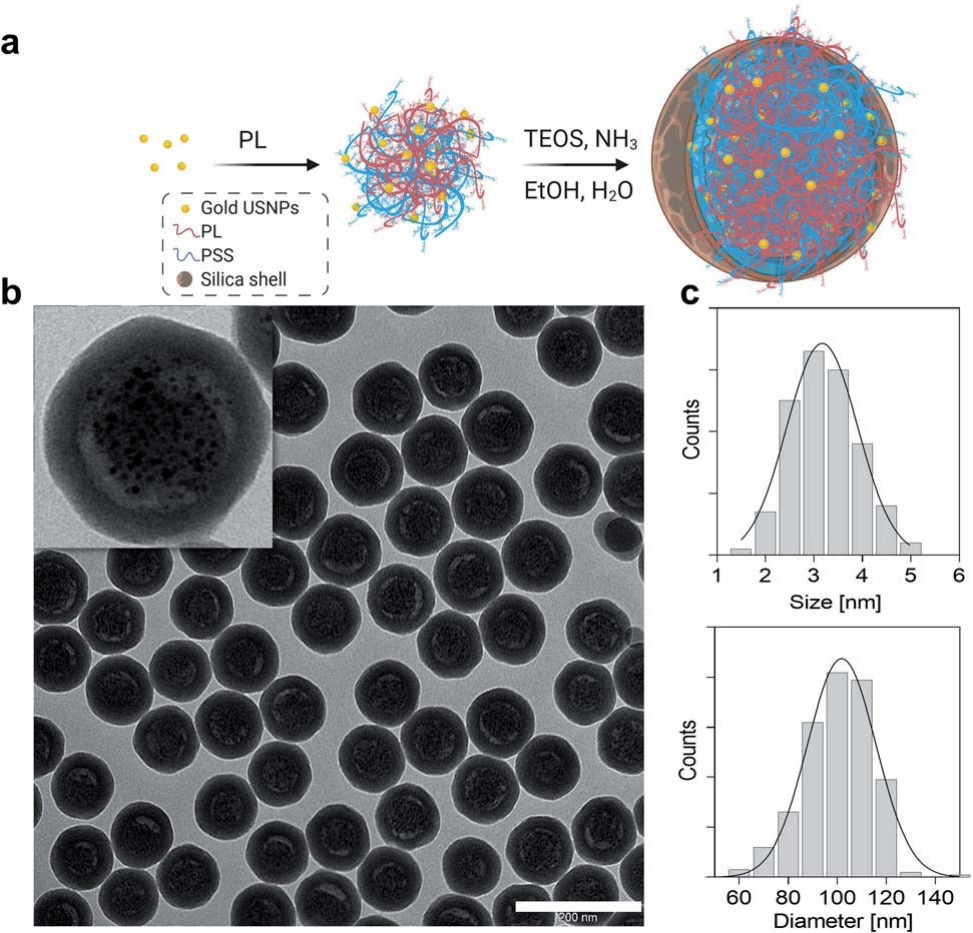
(a) Scheme for the synthesis of NAs. Gold seeds are synthesized in the presence of PSS and assembled in controlled aggregates with poly(l-lysine) (PL). The aggregates are employed as templates for the formation of the silica nanocapsules. (b) Typical wide-area TEM image of NAs. Scale bar: 200 nm. Inset: zoomed-in image of one NA. (c) Size distribution of USNPs (upper) and NAs (bottom) based on at least 100 particles imaged by TEM.

Gold ultrasmall-in-nano architectures (NAs) were prepared following a standardized protocol.^19^ The gold ultrasmall nanoparticles (USNPs) contained in NAs were formed through fast reduction of gold using sodium borohydride in the presence of poly(sodium 4-styrenesulfonate) (PSS), resulting in USNPs with an average diameter of 3.2 ± 0.7 nm (Fig. 1). The ionic interaction between poly(l-lysine) and PSS-coated USNPs supported a controlled aggregation, and the resulting polymeric construct served as a template for the formation of the silica shell. The final NAs have an average diameter of 101.8 ± 13.9 nm (Fig. 1) and a metal loading of 4.5% w/w. As previously demonstrated, the hydrodynamic diameter of NAs in blood-mimicking solution is not substantially altered over 8 h.^20^ It is worth remembering that NAs (bio)degrade into their building blocks, *i.e.* silicic acid, polymers, and gold USNPs, within 48 h, as comprehensively investigated in various relevant physiological fluids (human serum and blood) and a cellular environment (Fig. S1).†^14,21^ Furthermore, NAs demonstrate encouraging clinical translation potential for a noble metal-based nanomaterial, due to their biosafety and non-persistent behaviours.^18,20^ Some potential biomedical applications of NAs have been investigated and resulted from the joint presence of all components.^12,13,22^

Briefly, the hollow silica shell is conceived as a shielding nanocapsule that (i) preserves the encapsulated materials until its degradation, (ii) enhances ultrasound echo signals and (iii) offers a straightforward modifiable surface.^12,23^ Meanwhile, the gold USNPs promote light–matter interactions essential for photothermal effects or for radiosensitization, while the polymer can be modified with active molecules, such as drugs and dyes.^13,21,22^

**Fig. 2:**
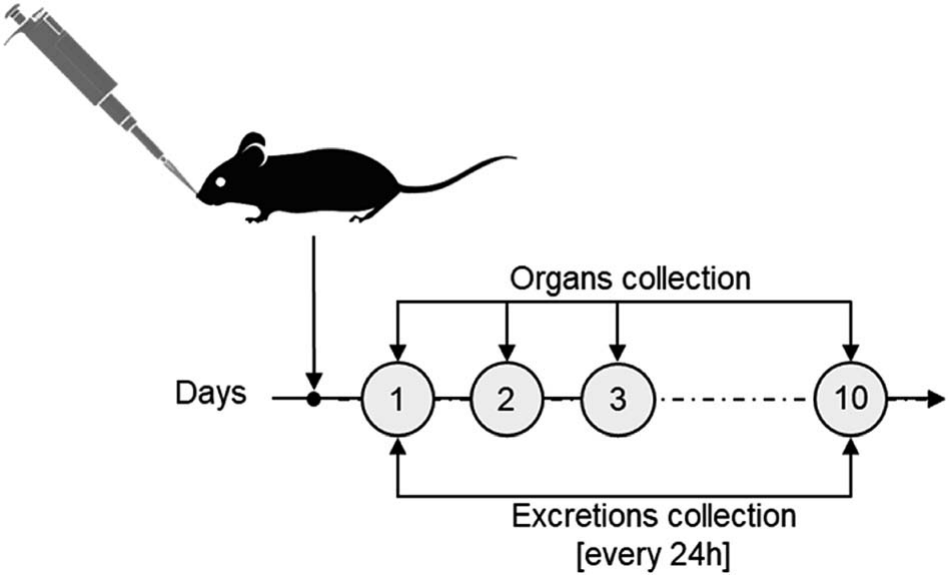
Experimental scheme for the *in vivo* NA biodistribution and excretion assessments.

The biokinetics and excretion of NAs after intravenous (IV) tail-vein administration was previously investigated on CD1-Foxn1^nu^ mice models, a species generally employed as an orthotopic and heterotopic xenograft tumour model.^20^ In particular, we confirmed a good excretion of gold in 10 days associated with a drastic reduction of its presence in the liver.^20^ For consistency, the same mouse model was employed in this study on intranasal (IN) administration. IN administration, an accepted procedure in pharmacology and toxicology investigations, was preferred over intratracheal (IT) instillation or nebulization (N) as it is less invasive than IT and delivers more accurately compared to N.^10^ Each mouse (average of 30 g per model) was treated with 3 mg NAs per kg mouse, corresponding to approximately 4 μg of gold, a lower amount than the upper limit suggested for inhalation experiments on rodents.^24^ After IN administration, urine and faecal excretions were collected daily for 10 days, and mice were sacrificed at 4 time points (Fig. 2). The time points were chosen considering the (bio)degradation timeframe of NAs and the reduction of potential stress on the mice, which were single-housed in metabolic cages in order to collect excretion preventing their cross-contamination. Potential behavioural abnormalities, pathological signs, and mortality in treated mice were monitored for the whole experimental period. During the course of the exposure, no adverse effects on animals were observed upon daily inspection. In particular, no significant variations in body weight, food consumption, and water intake among the animals were observed after NA administration (Fig. S2).† The biodistribution of gold was quantitatively determined by ICP-MS analysis of organs and excretions. It has already been demonstrated that the amount of gold was negligible in mice not treated with NAs. Thus, in agreement with the 3R's concept, experiments on untreated mice were omitted.^18^ All data in this manuscript are reported in % injected dose (% ID) to provide a direct overview of the gold biodistribution with respect to the administered amount. For further information, all data are also reported in terms of detected gold (μg) and % ID g^−1^ in the ESI† (Fig. S3 and S4†).

**Fig. 3:**
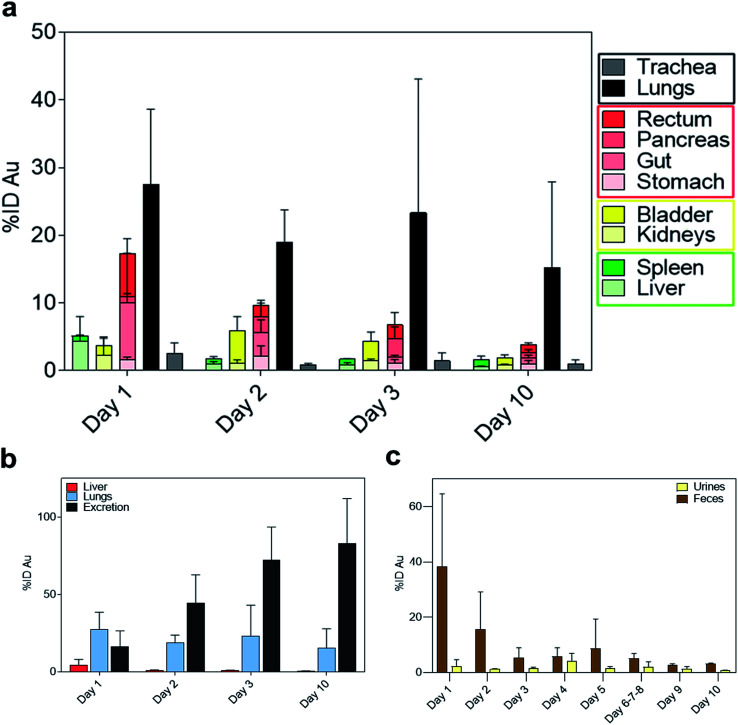
(a) Gold biodistribution (%ID) in the main organs grouped by body systems (respiratory system in black, digestive system in red and excretory systems in yellow and green, respectively) over 10 days. (b) %ID of gold in the liver (red), lungs (light blue) and cumulative urine and faeces (black). (c) Daily distribution of gold determined by ICP-MS in urine and faeces during 10 days after the administration of NAs (*n* = 3). Results are reported as mean ± standard deviation.

Interestingly, on day 1 after IN administration, a significantly higher amount of gold was detected in the lungs compared to the trachea (Fig. 3a). This confirms that NAs reached the lower airways avoiding accumulation in the upper region. The pulmonary localization of NAs further demonstrated their potential ability to produce enhanced effects in the lungs.^25^ Substantial amounts of gold were also observed in the gastrointestinal (GI) organs (Fig. 3a). Indeed, a part of the administered bolus was ingested by the animals during the application process. Meanwhile, the measured amount of gold in the liver was 4.37% ID on day 1 (Fig. 3b), which is markedly lower compared to IV administration (Fig. S5).† Overall, the biodistribution profiles are significantly different depending on the mode of administration.^20^

**Fig. 4:**
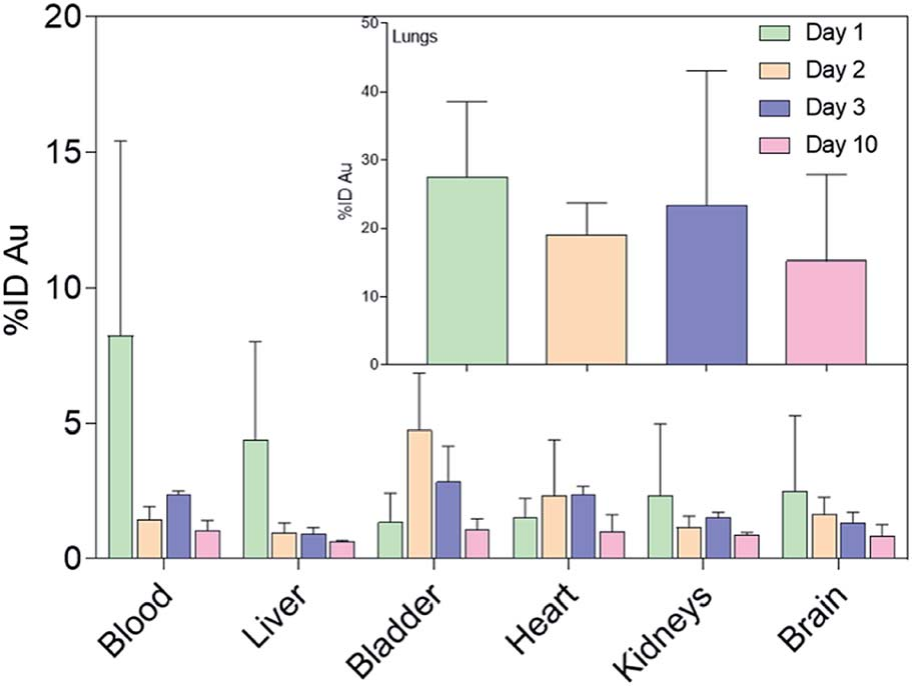
Gold biodistribution assessment (% ID) in the main vascularized organs. Inset: Gold biodistribution in the lungs. Results are reported as mean ± standard deviation.

The amount of gold collected in all the organs gradually decreased until day 10, while the cumulative excretion finally reached around 80% ID, confirming the non-persistence of NAs in the organism. In particular, the daily excretion of gold passed from around 1.62 μg on day 1 to around 0.16 μg on day 10 with an average of 3.32 μg excreted within the 10 day observation period (Fig. S6).† Gold was mainly recovered in faecal rather than urine samples, in contrast to the excretion behaviour of IV-administered NAs, wherein gold was mainly collected in urine samples (Fig. 3c and S7†).^20^ The decreasing trend in faeces within the first 3 days could be related to the excretion of NAs that were unavoidably ingested by the animal models. This also coincided with the decrease in the amount of gold in the GI tract. The presence of gold in the GI organs could also be attributed to the mucociliary escalator clearing mechanism. This process involves the entrapment of insoluble particulates in the gel layer of the mucus, directing them to the pharynx by the upward motion in the mucus, ultimately leading the particulates to the gastrointestinal tract.^2,3^ The strong involvement of the GI tract in faecal excretion was further validated by the low amount of gold in the liver, suggesting a less pronounced contribution of the hepatobiliary system. It was already demonstrated that gold USNPs resulting from the (bio)degradation of NAs can be excreted through the renal pathway; thus the presence of gold in urine was not surprising.^18,20^ Additionally, the amount of gold in the urine samples peaked on day 4 (Fig. 3c). This followed the maximum amount of gold collected in the bladder on day 2 (Fig. 4), which can be associated with the (bio)degradation of NAs, and perhaps with the translocation of gold from the lungs.

Particulates that bypass the mucus entrapment and enter the periphery of the lungs can be cleared through slower processes. These include immune system facilitated clearing mechanisms involving alveolar macrophages and dendritic cells, and translocation in the alveolar epithelium to the blood circulation.^3^ Since the mice employed in this investigation are immunodeficient, the excretion of nanomaterials in the lung parenchyma was limited to translocation.^26^ Consequently, significant amounts of gold were also measured in secondary organs (Fig. 4). For instance, the measured gold in the heart might have resulted from translocation from the lungs, as the nanoparticles crossed the air–blood interface and entered systemic circulation.^27^ Indeed, a significant amount of gold was also observed in the blood mainly on day 1. These outcomes also suggest a potentially crucial association between inhaled nanoparticles and cardiovascular disease, which needs to be considered in risk assessment and management of the use of engineered nanomaterials.^28^

A notable amount of gold (2.5% ID) was also found in the brain on day 1, which was approximately an order of magnitude higher with respect to the measured value after IV administration (Fig. S5).† This finding is of special interest as nanoparticle delivery to the brain is particularly challenging because of the blood–brain barrier.^3^ The translocation of NAs to the central nervous system may have resulted from the involvement of the olfactory mucosal and neuronal pathway.^29,30^ Indeed, the size of NAs is <200 nm and they can pass through the cribriform plate, where the olfactory nerves pass from the nasal cavity to the olfactory bulb.^29,31^ Moreover, the amount of gold significantly decreased during the 10 days, confirming the ability of the building blocks of NAs to escape the brain and prevent prolonged organ persistence.

## Conclusions

In summary, we have demonstrated that gold ultrasmall-in-nano architectures are able to accumulate in the lungs after inhalation, translocate to secondary organs, and be almost completely excreted within 10 days. The nanoparticles were excreted mainly through the faeces, due to the mucociliary escalator clearing mechanism and the involvement of the gastrointestinal tract. On the other hand, the nanoparticles also passed through the air–blood interface and translocated to systemic circulation as evidenced by the gold detected in the blood and heart. Interestingly, NAs were also able to accumulate in the central nervous system through the olfactory neuronal pathway and escape the brain after (bio)degradation.

This work paves the way for the development of systemic or local pulmonary-delivered noble metal-based treatments for oncology and infectious diseases. Notably, investigations on the fate of well-quantifiable materials may also provide interesting insights into involuntarily inhaled nanomaterials.

## Materials and methods

### Materials

All chemicals were purchased from Sigma-Aldrich, unless otherwise specified, and used as received.

### Synthesis of gold ultrasmall nanoparticles and polymeric arrays

Gold ultrasmall nanoparticles (USNPs) were prepared by adding 200 μL of aqueous solution of HAuCl_4_ (stock: 10 mg mL^−1^) and 10 μL poly(sodium 4-styrenesulfonate) (70 kDa, 30% aqueous solution, PSS) into 20 mL of Milli-Q water. The chloroauric acid solution was stirred vigorously and 200 μL of sodium borohydride (stock: 8 mg mL^−1^) was quickly added. The solution continued to stir vigorously for 2 minutes and was aged for 10 minutes. Then, 75 μL of aqueous poly(l-lysine) (stock: 40 mg mL^−1^) was added for controlled aggregation of the PSS-coated Au USNPs. The solution was further incubated for 20 minutes. The Au polymeric arrays were collected by centrifugation at 14 000 rpm for 5 minutes and then resuspended in 2 mL Milli-Q water.

### Synthesis of nano-architectures

A modified Stöber process was employed to allow silica shell formation in the periphery of Au polymeric arrays. Here, two 50 mL tubes were each filled with 35 mL of ethanol, 1.2 mL ammonium hydroxide solution (30% in water), 20 μL tetraethyl orthosilicate (TEOS, 98%), and 1 mL of the Au polymeric arrays. The reaction was allowed to proceed with moderate shaking for 3 hours. Then, the tubes were centrifuged at 4000 rpm for 30 minutes. After discarding the supernatant, the precipitates containing the nano-architectures (NAs) were washed by resuspension in ethanol, sonication, and centrifugation at 14 000 rpm for 3 minutes. The washing was discarded and another round of re-suspension–sonication–centrifugation was performed. Then, a short spin (15 seconds or until the rotational speed reaches 14 000 rpm) was performed to remove bigger NAs. The supernatant was again spun for 3 minutes at 14 000 rpm to collect the nano-architectures. Finally, the NAs were resuspended and stored in ethanol (1 mL).

### Electron microscopy

The nano-architectures were sonicated to homogenize them and 5 μL was deposited onto a 300-mesh carbon-coated copper grid. Transmission electron microscopy images were taken using a ZEISS Libra 120, operated at 120 kV accelerating voltage.

### Inductively coupled plasma-mass spectrometry (ICP-MS) measurements

A small volume (*e.g.* 5–10 μL for 1× synthesis) of NAs was placed in a 10 mL pressure vessel, and freshly made aqua regia prepared using a 3 : 1 ratio of ICP-MS grade hydrochloric acid and nitric acid was added. The vessel was sealed and placed in a CEM Discover SP-D for digestion under microwave irradiation (200 °C/15 minutes). The digested sample was diluted to a final volume of 5 mL with ICP-MS grade water.

Samples of mice organs and excretions were first dried overnight at 80 °C until a constant weight was obtained. Dried samples were transferred to 10 mL pressure vessels and digested in nitric acid (∼3 mL) at 150 °C for 30 min on a hot plate. The acid was allowed to evaporate before performing another round of digestion using freshly made aqua regia at 150 °C for 30 min. Finally, samples were dried and diluted to a final volume of 5 mL with 3% nitric acid solution.

The amounts of gold were determined after analysis on an ICP-MS Agilent 7700, using standard calibration curves.

### *In vivo* experiments

Male CD1-Foxn1^nu^ mice, 9 weeks old (Charles River, Calco, Italy), were used for *in vivo* tests. The animals were group-housed in ventilated cages and had free access to food and water. They were maintained under a 12 hour light/dark cycle (lights on at 8:00 am) at a controlled temperature of 21 ± 1 °C and relative humidity of 55 ± 10% for one week. After acclimation, the animals were housed singularly in metabolic cages, in order to collect urine and feces, after 1, 2, 3 and 10 days (*n* = 3 mice per group). All experiments were carried out in accordance with the guidelines established by the European Communities Council Directive (Directive 2010/63/EU of 22 September 2010) and approved by the National Council on Animal Care of the Italian Ministry of Health. All efforts were made to minimize animal suffering and to use the minimal number of animals required to produce reliable results. Only for the experimental period were the mice housed in metabolic cages and monitored daily for body weight, food and water intake, and urine and feces output.

NAs were resuspended in sterile saline to reach the final concentration of 3 mg NAs per kg mouse. CD1-Foxn1^nu^ male mice were anesthetized with ketamine and xylazine (100 and 10 mg kg^−1^, respectively, i.p.). Mice were intranasally administered with 20 μL of NAs dissolved in saline into both nares by using a pipette. At different time points (1, 2, 3 and 10 days), mice were sacrificed and tissues collected and snap-frozen in liquid nitrogen and stored at −80 °C until required for subsequent analysis.

## Conflicts of interest

The authors declare no conflicts of interest.

## Supplementary Material

NA-002-D0NA00521E-s001
