# Supplementary material for: Biokinetics and clearance of inhaled gold ultrasmall-in-nano architectures
Source: Nanoscale Adv. 2020 Jul 22;2(9):3815–20. doi: 10.1039/d0na00521e (PMC9417912; doi:10.1039/d0na00521e)
Supplement: NA-002-D0NA00521E-s001 [file NA-002-D0NA00521E-s001.pdf]

Electronic Supplementary Material (ESI) for Nanoscale Advances.
This journal is © The Royal Society of Chemistry 2020

SUPPORTING INFORMATION

Biokinetics and clearance of inhaled gold ultrasmall-in-nano architectures

Ana Katrina Mapanao,[#,1,2] Giulia Giannone,[#,1,2] Maria Summa,[#,3] Maria Laura Ermini,[1] Agata Zamborlin,[1,2] Melissa Santi,[1] Domenico Cassano,[1] Rosalia Bertorelli,[3] and Valerio Voliani[*,1]

[1.] Center for Nanotechnology Innovation@NEST, Istituto Italiano di Tecnologia, Piazza San Silvestro, 12 - 56127, Pisa, Italy

[2.] NEST-Scuola Normale Superiore, Piazza San Silvestro, 12 - 56127, Pisa, Italy

[3.] Translational Pharmacology, Istituto Italiano di Tecnologia, Via Morego, 30 - 16163, Genoa, Italy

[#]these authors have contributed equally

*Corresponding Author: valerio.voliani@iit.it

Supplementary Figures and Tables

Figure S1

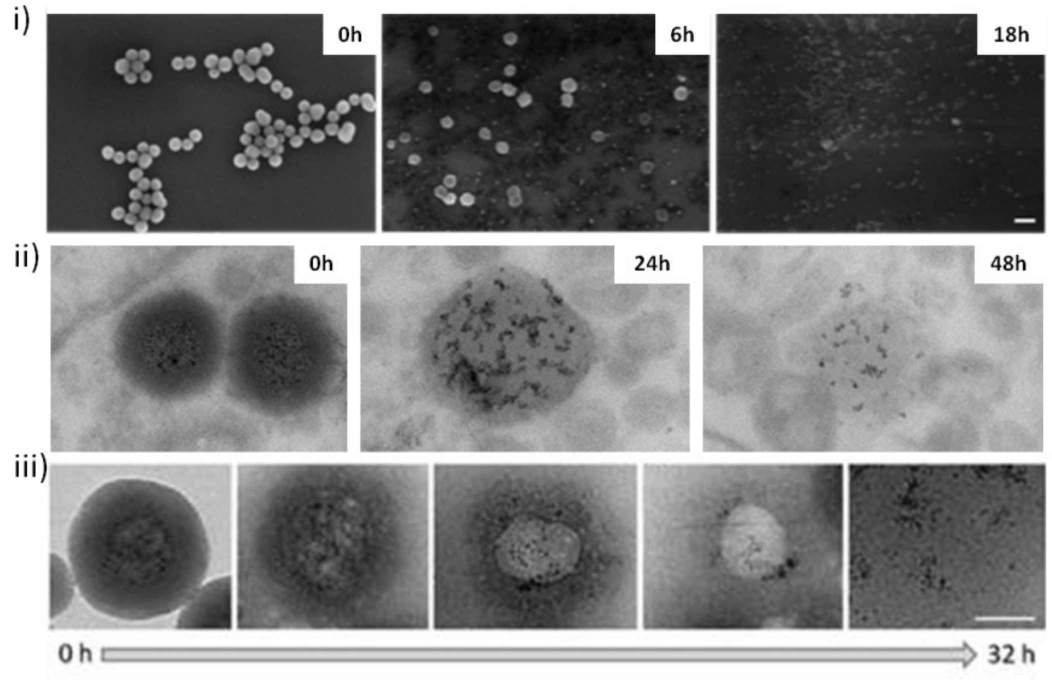

Figure S1 Electron microscopy imaging of NAs biodegradation in biological environment. i) 20% full human serum at 37 °C under stirring. ii) 2D-cultured MIA PaCa-2 cells. iii) full human blood at 37 °C under stirring.

Figure S2

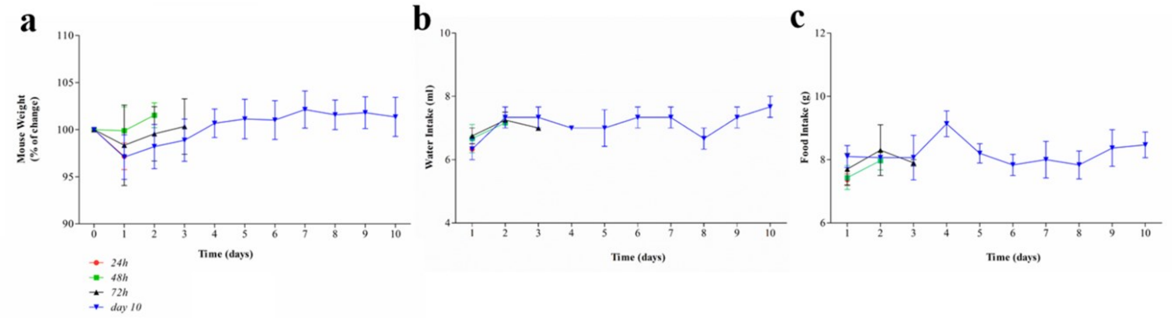

Figure S2 Body weight (a), Water intake (b), and Food intake (c) of the animal models after inhalation of NAs. No significant changes were observed in all measured parameters.

Figure S3

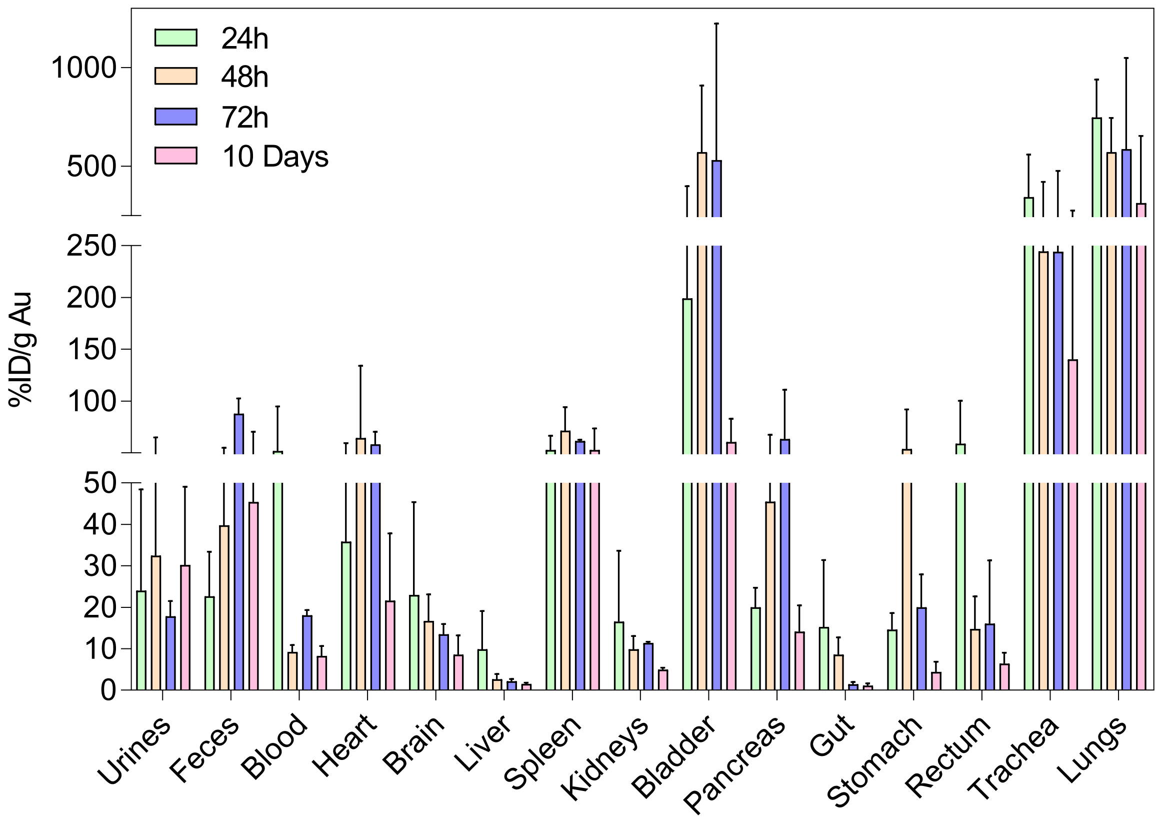

Figure S3 Gold biodistribution (%ID/g) in organs and excretions (urine and feces) determined by ICP-MS at the selected time points (n=3).

Figure S4

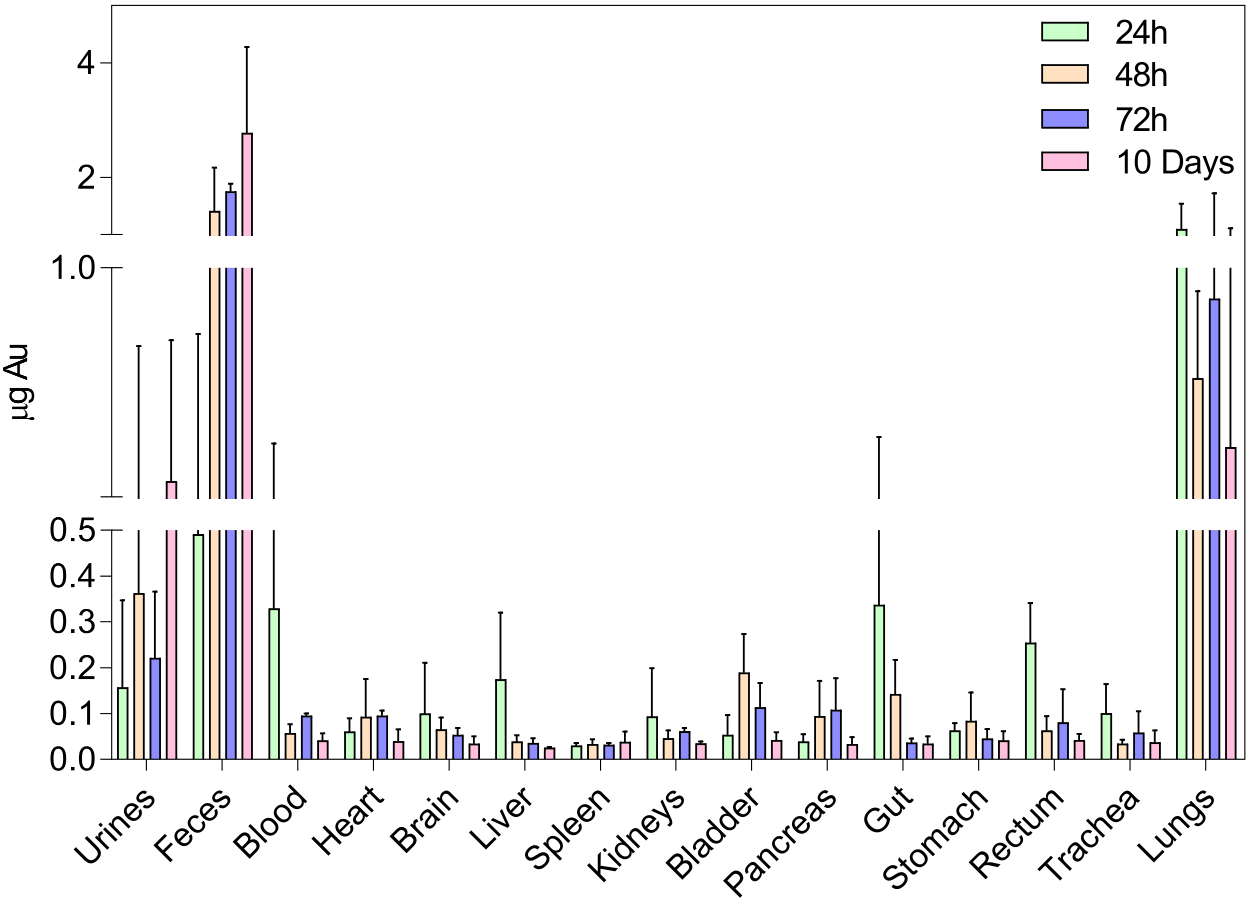

Figure S4 Biodistribution (µg) of gold in organs and in the excretions (urine and feces) determined by ICP-MS at the selected time points (n=3). The cumulative amount of gold detected was 3.8 ± 1.0 µg.

Figure S5

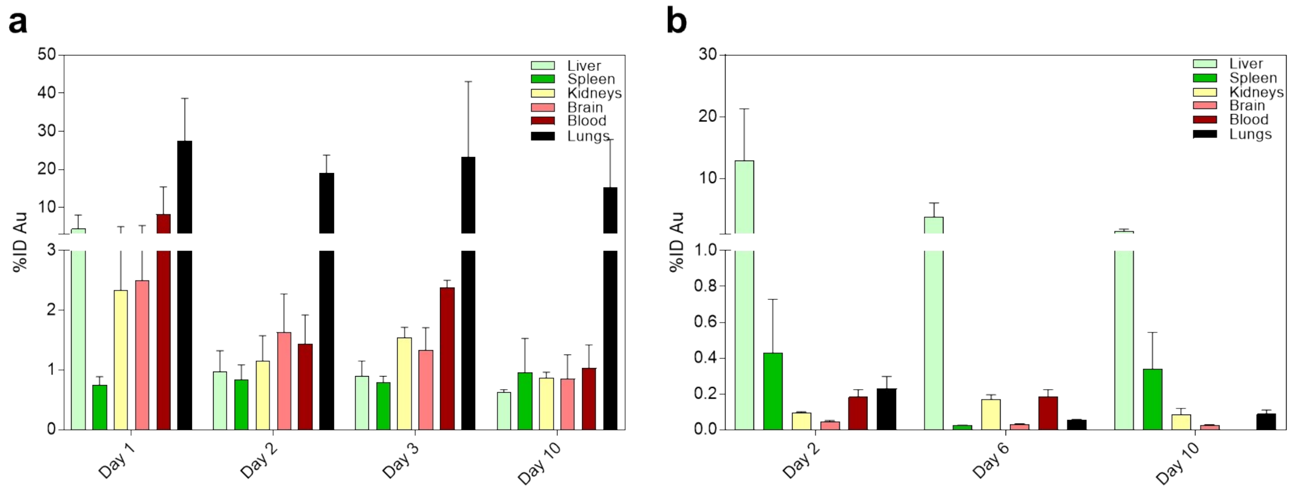

Figure S5 Gold biodistribution assessment (%ID) in the main organs over 10 days. (a) IN administration (b) IV administration

Figure S6

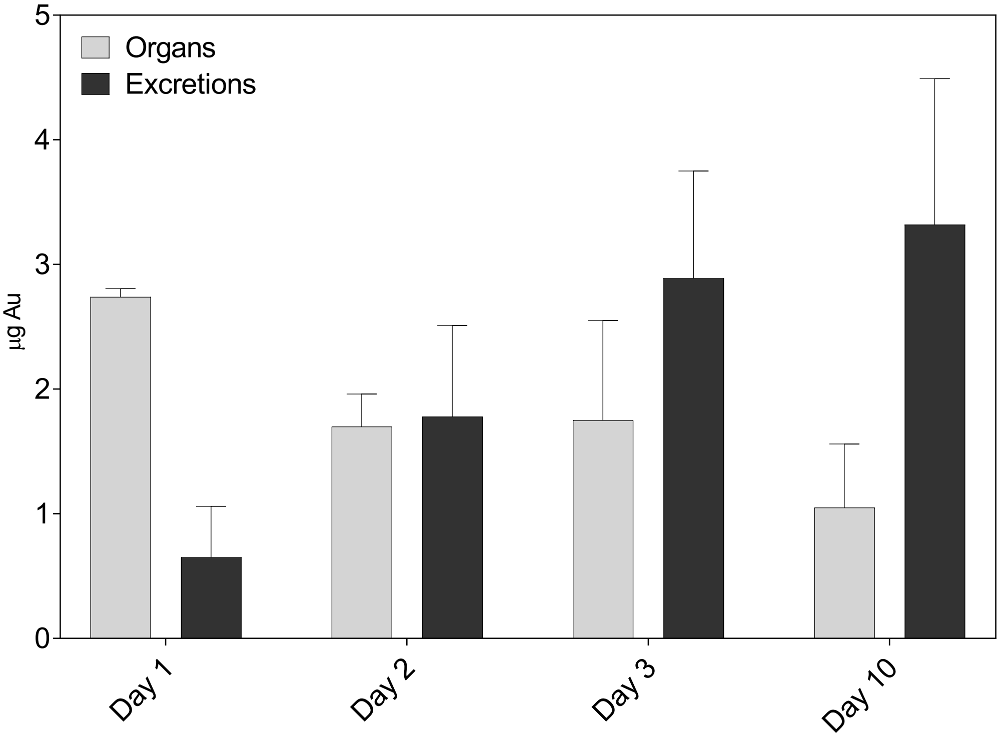

Figure S6 Amount (µg) of gold in organs (cumulative) and in the excretions (urine and feces, cumulative) determined by ICP-MS at the selected time points (n=3)

Figure S7

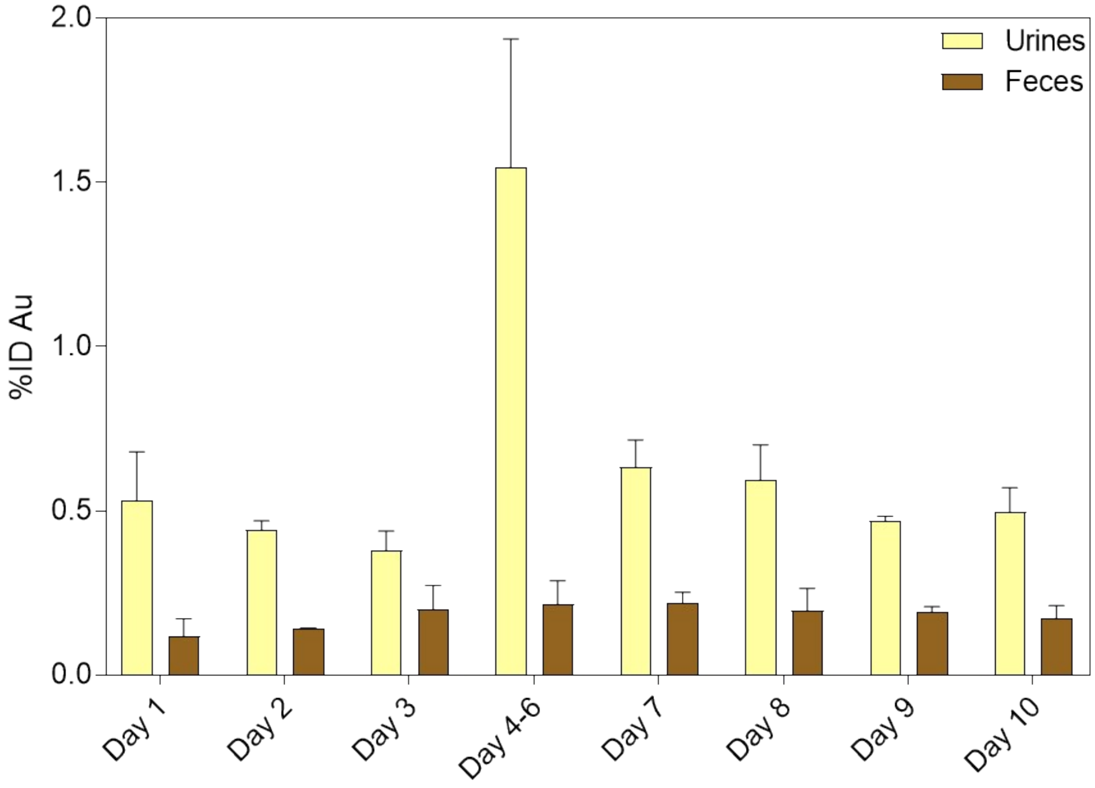

Figure S7 Daily distribution of gold determined by ICP-MS in urine and feces during 10 days after the intravenous administration of NAs
